# An Overview of the Sustainable Depolymerization/Degradation of Polypropylene Microplastics by Advanced Oxidation Technologies

**DOI:** 10.3390/molecules29122816

**Published:** 2024-06-13

**Authors:** Elisa I. García-López, Narimene Aoun, Giuseppe Marcì

**Affiliations:** 1 Department of Biological, Chemical and Pharmaceutical Sciences and Technologies (STEBICEF), University of Palermo, Viale delle Scienze, 90128 Palermo, Italy; elisaisabel.garcialopez@unipa.it; 2 Department of Engineering (DI), University of Palermo, Viale delle Scienze, 90128 Palermo, Italy; aoun.narimene95@gmail.com

**Keywords:** depolymerization, microplastics, advanced oxidation technologies (AOTs), sonochemistry, ozonation, photocatalysis

## Abstract

Plastics have become indispensable in modern society; however, the proliferation of their waste has become a problem that can no longer be ignored as most plastics are not biodegradable. Depolymerization/degradation through sustainable processes in the context of the circular economy are urgent issues. The presence of multiple types of plastic materials makes it necessary to study the specific characteristics of each material. This mini-review aims to provide an overview of technological approaches and their performance for the depolymerization and/or degradation of one of the most widespread plastic materials, polypropylene (PP). The state of the art is presented, describing the most relevant technologies focusing on advanced oxidation technologies (AOT) and the results obtained so far for some of the approaches, such as ozonation, sonochemistry, or photocatalysis, with the final aim of making more sustainable the PP depolymerization/degradation process.

## 1. Introduction

**Figure 1:**
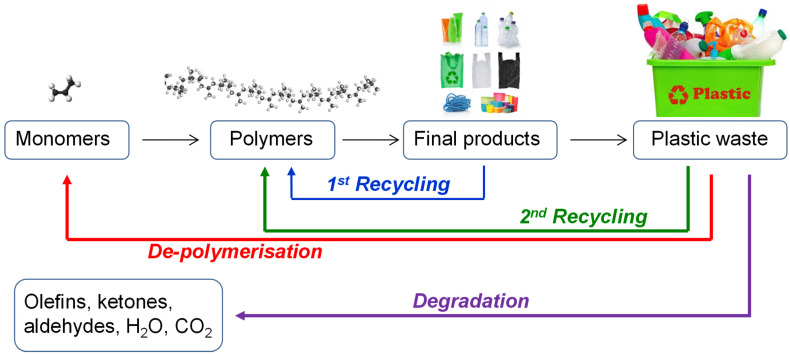
Scheme of the plastics waste recycling process or plastics degradation to obtain olefins, ketones, aldehydes with low molecular weight, and water and carbon dioxide.

The ubiquitous presence of solid plastics is an issue of growing environmental concern. A world without plastics seems today unimaginable, indeed, the increase in plastics production has been remarkable, surpassing most other manufactured materials, with the possible exception of steel or cement. The number of plastics in municipal solid waste was 1% in mass in 1960, increasing to more than 10% by 2015 [1]. Plastic waste is so ubiquitous in the environment that it has been suggested as a geological indicator of the proposed Anthropocene era [2]. The majority of the monomers used for the predominantly used plastics are derived from fossil hydrocarbons and they are not biodegradable; consequently, they accumulate in the natural environment [3]. Currently, the only strategy to permanently eliminate plastic wastes is combustion or pyrolysis. An amount of 14% of the plastic packaging is collected for recycling and 2% of all recovered plastic packaging waste returns to applications of the same or similar quality (primary recycling), whereas the remaining plastic packaging waste escapes into the environment during transport, use, or end-of-life collection failures [4]. Plastic debris has been found in all major ocean basins. The recycling of polymers necessitates different processes, such as the separation of impurities and the degradation of macromolecular structures, which influence the properties of the recycled materials (secondary recycling). A great challenge for polymer chemistry would be to develop stratagems where the wasted polymers could be transformed into their own starting materials, i.e., transform the current polymers, ubiquitously present as a waste, back into monomers, and purify them for re-polymerization. The transformation of polymers back into monomers (de-polymerization) is a process of great environmental value because the material recycled in this manner does not lose properties and would raise the ideal circular economy. Many research efforts are currently devoted to polymer recycling, although still, few investigations obtain successful results in which depolymerization gives rise to a monomer, or inferior fragments of the original polymer, possessing sufficient qualities to obtain the original polymer with the same initial properties, i.e., without suffering any loss of functionality. An alternative to reduce the environmental impact of plastic wastes is their degradation process, which should give rise to the complete breakage of C-C bonds in order to obtain H_2_O and CO_2_ as the final products. However, the potentially dangerous chemical substances released during the degradation of the polymers are aspects of huge environmental interest, and, consequently, their de-polymerization for the reinsertion in the cycle economy, as schematized in Figure 1, is preferable.

**Scheme 1:**
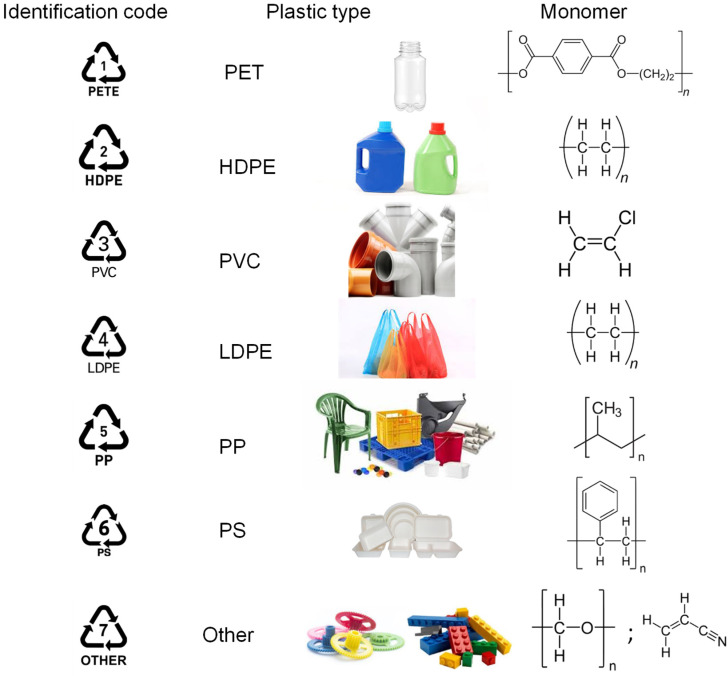
List of most common plastics, their constitutive monomer, and identification code.

Plastics are classified into seven categories based on the degree of hazard to humans and the environment, as well as considering their recyclability. The most commonly used plastic materials are reported in Scheme 1. Polyethylene terephthalate (PET) is one of the most produced types of plastic in the world. It is commonly used for packaging or beverage bottles. The second kind of plastic is polyethylene (PE), which is divided into two groups, i.e., HDPE (high density) and LDPE (low density). HDPE is a rigid plastic used for robust plastic packaging, such as laundry detergents, as well as construction applications or trash cans. LDPE is a transparent and flexible plastic used for plastic bags. It is highly flexible but presents low tensile strength. PE shows corrosion resistance. Another type of PE is the high molecular weight PE, which can be even stronger than steel, and it is mostly used in medical devices such as pelvic implants. Polyvinyl chloride (PVC) is a transparent and impact-resistant plastic, mostly used in construction and commercial applications such as plumbing, electrical wire insulation, and strong packaging. PVC is difficult to recycle and less than 1% is recycled. Polypropylene (PP) is a durable semi-transparent plastic with a low friction surface. It does not react with liquids and possesses good electrical resistance. The most widely used plastic on the market is PP due to its high flexibility and compatibility. Polystyrene (PS) is a versatile plastic applied in disposable tableware, building insulation, and as a transparent material or medical devices such as test tubes or Petri dishes. Finally, there are a series of polymers, indicated as type 7 in Scheme 1, which are polycarbonate (PC) and other plastics, such as polyoxymethylene (POM), polylactic acid, nylon (or polyamide), polymethyl methacrylate (PMMA), acrylonitrile butadiene styrene (ABS), among others [5,6].

Polyethylene (PE) and polypropylene (PP) are the largest group of commercial synthetic plastics and by far the most important polymers. Its application in packaging is massive, as it is the second most used plastic in the world [7]. These materials are polyolefins, possessing the general formula (-CH_2_CHR-)_n_ where R is a hydrogen or a methyl group. PP emerges for its great mechanical resistance, lightness, excellent electrical insulation ability, and inertness to water. Both polyolefins are highly stable and do not readily degrade in the biosphere, so their massive waste quantities contaminate enormously. It is alarming to know that of the 8.3 billion metric tons of plastics manufactured since the 1950s, almost 80% have become waste [8]. The increased production and use of plastics gave rise to accumulation in marine, freshwater, and terrestrial ecosystems. Indeed, the serious problem is related to the presence of microplastics, which cause pollution by entering natural ecosystems. Microplastics (MPs) are plastic pieces measuring less than 5 mm whose variable composition has been individuated in many environments. The most common are PE, PP, and PVC [9]. For instance, the microplastics used in personal care products are generally PE and PP, present in municipal wastewater treatment plants and ultimately in the environment. Microplastics are categorized as primary microplastics, the raw materials used in domestic and personal care products, and secondary microplastics arising from the degradation of raw plastic particles by physical, chemical, and biological processes in the environment [10]. Long-term durability due to their polymeric structure and easy transport between different habitats make microplastics of high concern. According to biologist studies, PP microplastics are more toxic than PE [11]. For instance, Schiavo et al. have compared the polyethylene (PE), polystyrene (PS), and polypropylene (PP) microplastics impact on the inhibition of marine microalgae *Dunaliella tertiolecta* population growth, DNA damage, and oxidative stress (ROS production), and they have classified the toxicity of polymer as follows: PP > PS > PE [12]. Other authors found PP more toxic than polyvinyl chloride (PVC) as regards mice pulmonary toxicity [13].

Technologies for the elimination of MPs from water and wastewater are urgently needed. The concern is enormous because polyolefins are bio-inert, and they are highly resistant to degradation by microorganisms such as fungi and bacteria. The surfaces made from polyolefins are hydrophobic and thus restrain the growth of microorganisms on their surfaces [14]. Several reviews summarized the microorganisms that can degrade plastics as PP, PE, and others, reporting the kind of microorganisms and enzymes and the metabolic pathways for plastic degradation [15,16]. The bio-deterioration of PP utilizing bacterial strains and complex microbial populace; however, has been reported to be very slow [17]. Microorganisms capable of biodegrading microplastics can give rise to certain depolymerization of PP into monomers or oligomers, but they can enter into the cells, so further efforts are needed both to understand the process and to induce further degradation.

All atoms of PE and PP are connected through strong single C-C and C-H bonds, and the chemical inertness of polyolefins sets a difficulty for their depolymerization by low energy processes. PP is less stable than PE because it has tertiary carbons, which are more sensitive to oxygen attack [18]. Its exposure to sunlight and the related degenerations are important subjects that have attracted scientific interest. Severe molecular chain degradation in PP can be induced when it is irradiated within the active wavelength range of 310–350 nm, which means that photodegradation can occur in PP-based materials [19], as explained below.

## 2. Methodologies Used for Depolymerization and Degradation of Polyolefins

**Figure 2:**
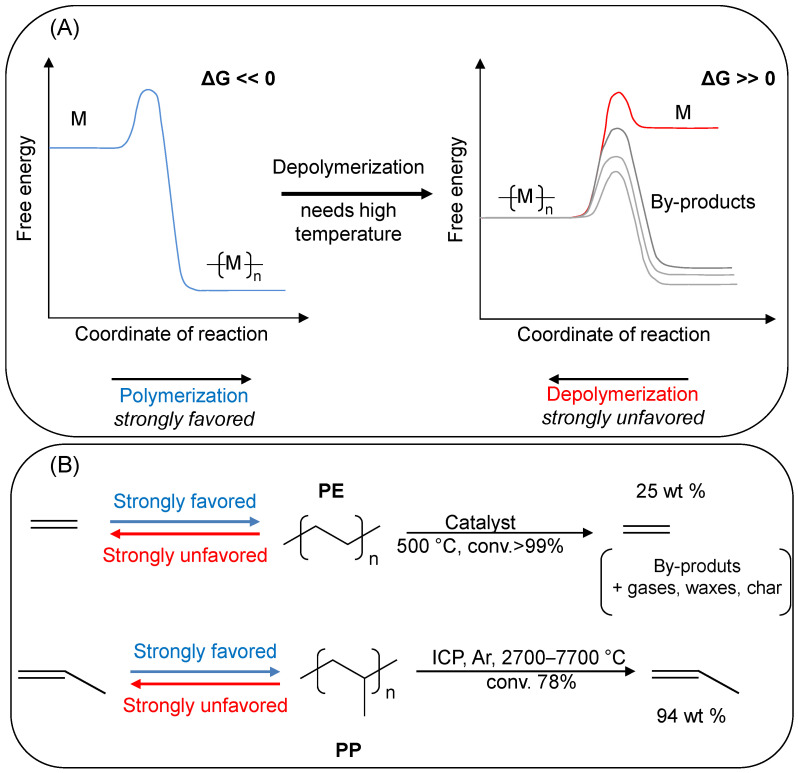
(**A**) Conceptual reaction profiles for a highly exergonic polymerization reaction and its corresponding depolymerization, where M represents the monomer. In these cases, the variation in Gibbs free energy is too negative for the reverse reaction of depolymerization. (**B**) Two representative examples of highly exergonic polymerizations, polyethylene (PE), and polypropylene (PP), and the correspondent depolymerization conditions and yield towards the original monomer [26].

The polymerization process of polyolefins is an exergonic process and, by virtue of the kinetic efficiency of the Ziegler–Natta industrial catalysts, their polymerization is rather economical. The polymerizations are exothermic enough to provide their own heat, so not consuming external energy (Figure 2). Successful depolymerization must activate the polymer chain and create reactive species capable of depolymerization. For most polymers formed by addition through a C-C π bond, selective reactivation for chemical recycling into monomer is thermodynamically and kinetically demanding under moderate conditions, hence polyolefins, such as polyethylene (PE) or polypropylene (PP), are emblematic in solving this problem (Figure 2). The long chains of the polymer molecules can be cracked by chemical or physical (or biological) processes, giving rise to short units of lower molecular weight [20].

Depolymerization is an important strategy in the framework of the circular economy, as schematized in Figure 1. Depolymerization differs from degradation, which involves the reduction of the molecular weight of the polymer along with its partial oxidation or, in some cases, the complete destruction of the chemical structure of the substance. On the other hand, depolymerization concerns only the reduction of the molecular weight of the polymer without major changes in the chemical structure [21].

Degradation during mechanical reprocessing is common for plastics. Polyolefins degrade during the melting process due to radical reactions that lower the molecular weight of PP [22]. Unless advances in recycling [23,24] of nascent purification technologies [25,26] or other research innovations alter this dynamic, polyolefin waste will continue to grow in proportion to polymer production. Unfortunately, monomer chemical recycling is not energetically accessible for polyolefins; in fact, according to Coates et al., chemical recycling of monomer for the three polymers produced in larger volumes, PVC, PE, and PP, is either chemically impossible, as in the case of PVC, or extremely challenging, as for polyolefins as PP or PE, as shown in Figure 2 [27].

Among the techniques investigated for polymer degradation, thermal, mechanical, photolytic, ultrasonic, microwave, biodegradation, oxidative, hydrolytic, and degradation by high-energy radiation are the most explored. Methodologies such as acid treatment, exposure to ionizing radiation, or enzymatic treatment suffer from major drawbacks such as higher treatment costs or uncontrolled reduction of the molecular weight, including changes in the chemical identity of the polymers [28]. Since all atoms in PP (and in PE) are linked via strong C-C and C-H single bonds, giving strong chemical inertness to the polyolefins, depolymerization by low-energy processes is a challenging procedure [29].

It can be mentioned that the generation of fuels from polyolefins has been extensively studied because of the absence of oxygen and the content of carbon and hydrogen in these materials, which, together with the absence of water in the polymer, confer the obtained fuels with a very high calorific value. Therefore, fuels produced from polyolefins possess similar combustible properties to fossil fuels and can become an alternative energy source [30].

**Figure 3:**
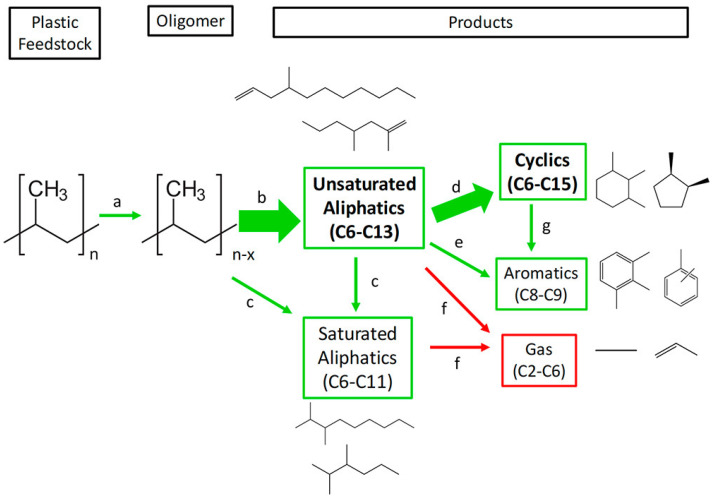
Scheme of the potential reaction of conversion of polypropylene by depolymerization process in supercritical water. A green box represents the oil phase products and a red box indicates the gas products. The thickness of the arrows represents the relative quantities of products. The letters on the arrows (a–g) indicate the evolution of the species. Reproduced from [32] with ACS permission.

The depolymerization of polyolefins in supercritical water has been also studied. This approach is a thermochemical process actuated at moderate temperature (>374 °C) and pressure (>22.129 MPa). Supercritical water gives rise to rapid, selective, and efficient reactions to convert organic waste into oil, compared to other depolymerization methods [31]. Recently, Chen et al. have converted polypropylene (PP) to oil in supercritical water for times in the range from 30 min to 6 h at 380–500 °C and 23 MPa [32]. A total of 91% of the weight of the PP was converted to oil at 425 °C in ca. 3 h or at 450 °C in ca. 1 h. At 425 °C, PP was rapidly (in ca. 30 min) decomposed into oligomers, and then the unsaturated aliphatics would be transformed into cyclics by cyclization. At the same time, small amounts of unsaturated aliphatics (olefins) can become saturated aliphatics (paraffins) and aromatics. Higher reaction temperatures (>450 °C) or longer reaction times (>4 h) lead to more gaseous products. About 80–90% of the oil components have the same boiling point range as naphtha (C_5_–C_11_) and heating values of 48–49 MJ∙kg^−1^. This conversion process is net energy positive and with higher energy efficiency and lower greenhouse gas emissions than incineration, mechanical recycling, or pyrolysis. Therefore, oil derived from PP has the potential to be used as gasoline blends or raw materials for other chemicals. The reaction pathway of the reaction process, as well as the main intermediates as proposed by Chen et al. [32], is reported in Figure 3.

### 2.1. Advanced Oxidation Technologies (AOTs)

**Figure 4:**
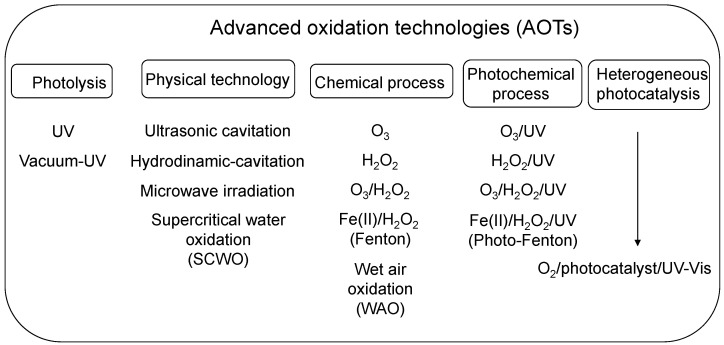
Different AOTs used for depolymerization/degradation of pollutants and chemicals recalcitrant to be depolluted.

Recently, effective results have been obtained by advanced oxidation technologies (AOTs). These methodologies are often used to mineralize organic pollutants and recalcitrant chemicals usually present in wastewater. AOTs utilize various oxidants such as H_2_O_2_, O_3_, or Fe^2+^ to produce reactive oxidizing species (ROS). The photo-chemical methods to degrade pollutants, including also PP degradation, are based on the production of ROS (^•^OH, ^•^O_2_^−^, ^1^O_2_, and h^+^) [33,34]. These species are strong oxidants and they can virtually oxidize any compound reacting unselectively with them once formed. The pollutant is speedily and efficiently fragmented and converted into small species. A series of processes can be considered AOTs, among them those summarized in Figure 4. The AOTs field has witnessed rapid development and, currently, Fenton, photo-Fenton, Electro-Fenton, and H_2_O_2_/UV systems as long as heterogeneous photocatalysis, particularly in the presence of TiO_2_, have received extensive scrutiny [34].

#### 2.1.1. Photolytic Degradation of Polymers

**Figure 5:**
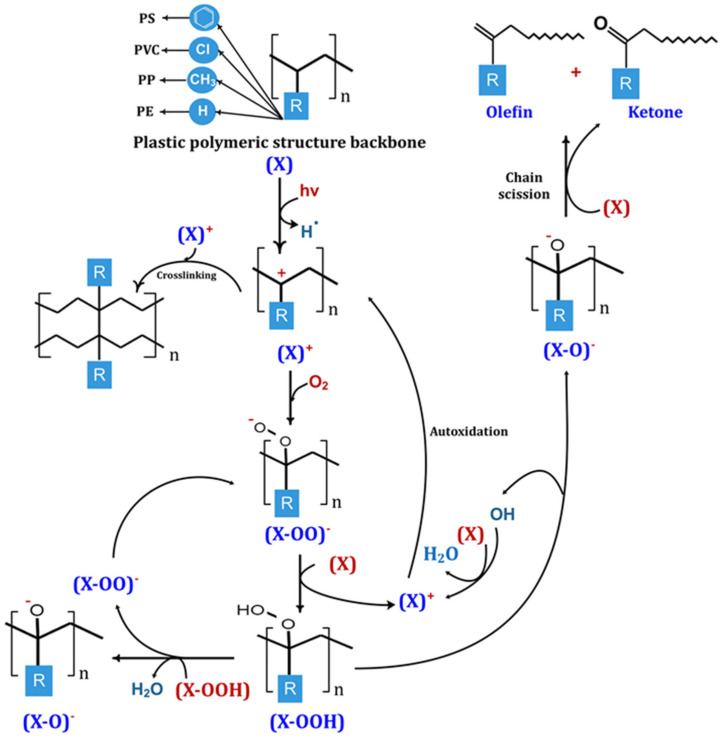
Mechanism of plastic UV photodegradation according to Ali et al. Reproduced with Elsevier permission from [37].

Degradation of plastic polymers in the natural environment can proceed by either abiotic or biotic pathways. Generally, abiotic degradation precedes biodegradation, and it is initiated thermally, hydrolytically, or by UV light [35]. PE and PP are susceptible to photo-initiated oxidative degradation, as schematized in Figure 5, which is believed to be their most important abiotic degradation pathway in aerobic outdoor environments [36]. Photodegradation in the absence of a catalyst (homogeneous) would be suitable to degrade polyolefins. This is the most important pathway of polyolefin abiotic degradation in aerobic external environments [37]. Photo-oxidation causes the oxygenation of the surface of the plastic, which increases the hydrophilicity of the polymer and improves the formation of the microbial biofilm on its surface. The reaction is divided into three main stages: initiation, propagation, and cessation. During the initiation phase, the chemical bonds of the polymer chain are cleaved by UV radiation to produce free radicals by breaking the C–H bonds. This process occurs only when polymers contain unsaturated chromophore groups that adsorb electromagnetic radiation, undergoing direct decomposition with bond dissociation upon ultraviolet [38]. As long as PP (and PE) does not contain unsaturated chromophore bonds, it is resistant to photo-initiated degradation. Nevertheless, the presence of impurities in the macromolecular structure can allow the photodegradation to proceed [38,39].

In the propagation phase, the polymeric radicals react with O_2_ forming ^∙^OOH radicals. In addition, further radical reactions give rise to the oxidation of the substrate [40]. Propagation eventually leads to chain splitting or crosslinking [41]. The combination of two radicals giving rise to inert species leads to the end of the reaction [42]. The oxidation gives rise to random chain cleavage producing oxygen-containing functional groups such as olefinic, ketone, and aldehyde compounds [39]. The formation of unsaturated double bonds during the process makes the molecules more susceptible to photodegradation. As the molecular weight of polymers decreases, the material becomes more susceptible to fragmentation through further reactions. For example, Albertsson et al. have studied the photodegradation of PE in an inert system for more than 10 years, showing that the degradation rate of PE is characterized by three phases. In the first stage, there is a rapid release of CO_2_ and absorption of O_2_ up to the equilibrium phase, while in the second stage, a decrease in the degradation rate is observed. Finally, a rapid deterioration of the surface structure and an increase in the degradation rate occurs [43].

As mentioned, traces of impurities in PP allow the formation of radicals that react with oxygen giving rise to radical reactions making random chain scission and crosslinking feasible, leading predominantly to lower molecular weight fragments [44]. Also, functional groups such as carbonyl and hydroperoxides are formed. The final species are pentane, 2-methyl-1-pentene, and 2,4-dimethyl-1-heptene, among others [45]. The biodegradability of a molecule is in relation to its chemical structure and chain branching increases the resistance to aerobic biodegradation; consequently, due to the presence of a tertiary carbon of PP, the predisposition to microbial degradation diminished.

**Figure 6:**
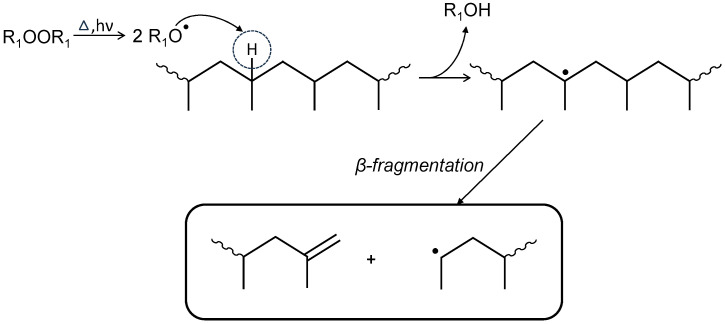
Polypropylene degradation in the presence of peroxide species by a β-fragmentation process.

The degradation of PP in the presence of peroxide both with and without contemporary light irradiation has been also examined. The degradation of PP depends on the quantity of O_2_, temperature, concentrations of both polymer and peroxide, and the radicals generated by the thermal decomposition of the peroxide [46]. Peroxides produce alkoxy radicals (highly reactive organic structures where the radicals are localized at the oxygen atom and singularly bound to an alkyl group) that abstract hydrogen atoms attached to tertiary carbons, and then the macro-radicals generated undergo β-fragmentation into alkenes and macro alkyl radicals (see Figure 6), which undergo further reactions.

**Figure 7:**
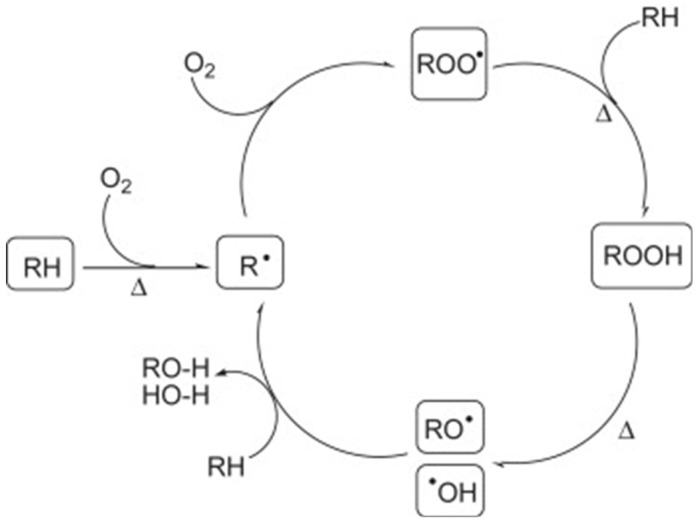
Oxidative cycle of polypropylene in the presence of oxygen, as proposed by Bertin et al. and reproduced with Elsevier permission [46].

Bertin et al. have observed that the extraction of H in the tertiary carbon is a minor process in the degradation of PP in the presence of peroxides in solution [46]; however, most of the literature reports the reaction pathway schematized in Figure 6. This is because the pathway depends very much on the experimental conditions and, in particular, on the presence of oxygen, the exclusion of which was difficult to achieve. Traces of oxygen increase PP degradation following the process schematized in Figure 7. Furthermore, degradation at high temperatures (about 300 °C) and the combination with cutting forces could also favor the fragmentation of the molecules. The reaction in the presence of peroxide radicals formed when O_2_ is present in the reacting medium follows the oxidative scheme of Figure 7. After its formation, the peroxy radical can undergo various reactions that lead to the shortening of the polymer chain.

The processes explained are also representative of the reactions that can be carried out in the laboratory in the presence of H_2_O_2_, O_3_, light, or ultrasound irradiation, as well as in the presence of solid acid catalysts such as silica-alumina or Nb_2_O_5_. The latter can be activated by the light generating further radicals through photocatalytic processes [47]. The addition of salts such as peroxodisulfate (S_2_O_8_^−2^) or Fenton reagent [48] can assist in the formation of ROS, which are extremely oxidant species, which will follow the same reactive pathway for PP degradation.

The influence of the temperature can be also considered because the thermal degradation of the plastic can be performed at temperatures above 100 °C, depending on the type and characteristics of the plastic polymer. The antioxidant additives incorporated through the manufacture of the plastic prevent thermal oxidation at low temperatures. Conversely, degradation due to thermal oxidation is accelerated by stress and exposure to reactive compounds such as O_3_ or H_2_O_2_ generated by ROS. In general, the resistance to degradation depends on the chemical composition of the polymer, with PP, PVC, and polybutadiene (PBD) being the most susceptible to thermal degradation. Conversely, polymers such as polysulfone, polyether ketone, and polysiloxanes are thermally resistant due to the strong bonds in their backbone. Overall, the contribution of thermal degradation under normal environmental conditions is considered to be globally negligible, particularly in cold marine environments.

#### 2.1.2. Ultrasound Irradiation (Sonochemistry)

Sonochemistry, based on the effect of ultrasound in forming acoustic cavitation in liquids, which results in a chemical activity has been extensively used for the degradation of pollutants, for instance, chlorinated organic compounds [49], or for the preparation of materials [50]. It is an eco-friendly green technology reported in many areas as organic chemistry, biomass valorization, electrochemistry, catalysis, environmental remediation, and also in polymer chemistry [51]. Ultrasound irradiation constitutes a valid alternative methodology for efficient depolymerization/degradation of PP [52]. It provides an accurate method to reduce the molecular weight of polymers in a targeted manner by carefully regulating both the rate of cleavage and the rate of polymerization. For the ultrasound-based approach, no chemicals are needed, so it offers a green alternative to other techniques; moreover, it can be modified by the presence of additives in combination with irradiation. Indeed, it has been reported that the use of additives and/or salts in combination with cavitation promotes the degradation of polymers [53].

The primary reactions that occur during sonication in water dispersion, which can be considered as the initiator of a series of radical reactions depending on the polymer species, are the following:

H_2_O→^•^OH + H^•^ (1)

H^•^ + H^•^→H_2_ (2)

^•^OH + ^•^OH→H_2_O_2_ (3)

H^•^ + O_2_→HO_2_^•^ (4)

H^•^ + HO_2_^•^→H_2_O_2_ (5)

HO_2_^•^ + HO_2_^•^→H_2_O_2_ + O_2_ (6)

Sonication in water gives rise to the formation of strong oxidizing agents like ^•^OH and H· radicals, which subsequently form hydrogen peroxide (H_2_O_2_).

A lower polymer concentration increases the degradation rate and produces a lower-weight molecule in shorter times because the overlap of the polymer chains decreases; therefore, they are more susceptible to the hydrodynamic forces generated by the cavitation forces. In the case of the cavitation reactor, the operating reaction volume plays an essential role in the extent of degradation. The polymer degradation rate decreased with an increase in the reaction volume. Consequently, the operating parameters strongly influence the extent of polymer degradation to maximize intensification.

Ultrasound has been applied for the degradation of various polymeric compounds such as polypropylene (PP) [54,55].

For the cavitation processes, it has been determined that (i) low concentrations and low volumes favor the degradation; (ii) type of polymer, in particular the presence of substituents or functional groups on the polymer chain, plays a significant role in the extent of degradation; and (iii) a reduction of viscosity occurred by increasing ultrasound frequency and power density up to an optimal limit for soluble polymers while only physical changes are observed for insoluble polymers. An optimal temperature must be established since it influences the process of initiation of cavitation and also the generation of free radicals based on the collapse of the cavity contents. The type of solvent plays also a crucial role in the overall effectiveness of the degradation process. The degradation rate mainly depends on the physico-chemical properties of the solvent such as volatility and kinematic viscosity. The degradation degree decreases with the increase in vapor pressure and viscosity of the solvent.

The presence of additives increases the effectiveness of the degradation of the polymer; for instance, a reduction of the viscosity occurs by adding salt up to an optimal value, while the effect of the addition of surfactants depends on the nature of the polymer. Of course, the addition of further oxidizing additives, such as ozone, increases the generation of ·OH, which can significantly increase the degradation of the polymer.

Chakraborty investigated the ultrasonic degradation of isotactic polypropylene at 80, 90, 113, 133, and 155 °C using o-dichlorobenzene as a solvent, using a frequency of 25 kHz with a voltage of 180 V [56]. By increasing the vapor pressure of the solvent and reaction temperature, the degradation rate decreased, even by decreasing the viscosity of the solvent. Price et al. studied the effect of irradiation intensity (26.2 ± 1.3 W·cm^2^) on solid polymer powder such as polypropylene suspended in water [57]. Particle fragmentation, deagglomeration, and surface modification resulted after the sonication treatment, and the extensions increased by increasing the irradiation intensity in a constant area of the transducer surface.

#### 2.1.3. Ozonation

Ozonation can be used as a process for the degradation of polymers; indeed, a sufficient amount of ozone can have a great effect on the degradation rate of polymers. Moreover, a synergistic effect has been observed in the rate of degradation by coupling ozonization with other advanced oxidation methodologies.

It is interesting to know that, according to Gugumus et al., the concentration of O_3_ in ambient air can range from 10 to 80 mg∙m^−3^ in winter and summer, respectively [58], so a seasonal influence on thermal oxidation of PP can be attributed to changes in ozone concentration in the natural ambient. To investigate this hypothesis on the thermo-oxidative degradation of PP films, the effect of lower O_3_ concentrations with respect to the environmental ones has been studied, concluding that an ozone concentration in the range of 100 to 200 mg∙m^−3^ does not affect the degradation rate of PP at a temperature of 120 °C [59]. Also, experiments carried out under an O_3_ flow twin-screw extruder with different polymer throughput and reaction temperatures demonstrate the ozone-thermal degradation of molten PP material on the reactive extrusion [60]. Ozone is introduced into the extruder to rapidly oxidize the molten PP within seconds. Oxidized PP had a higher melt flow index than the original PP, indicating a decrease in the molecular weight of PP. The ozone-induced degradation of PP may provide a way to produce PP with controlled rheology. These results indicate that O_3_ and temperature have a synergistic effect on the PP degradation reaction. Due to the fact that ozone is only in contact with molten PP for a few seconds, this process has higher reaction efficiency than solid-state PP degradation in an ambient containing ozone. It is worth noting that no harmful by-products are reported to be produced from the ozonating reaction [60].

**Figure 8:**
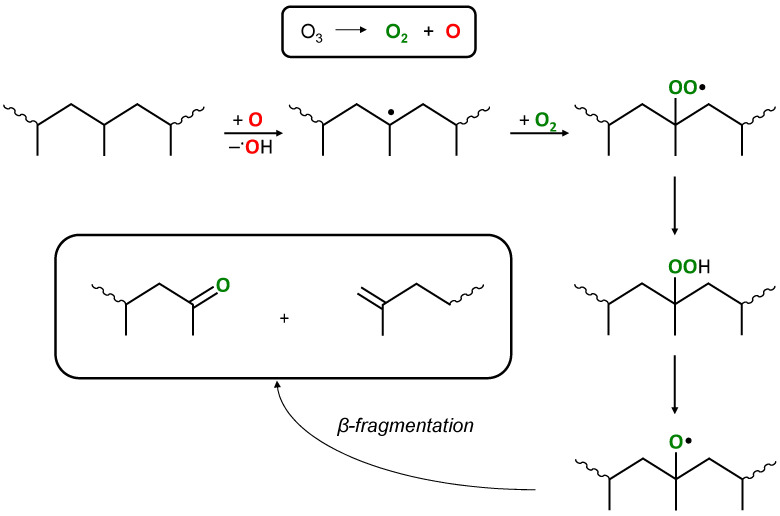
The mechanism of PP oxidative degradation with ozone.

The mechanism scheme of the oxidative degradation process of PP is shown in Figure 8. Atomic oxygen abstracted hydrogen atoms from the tertiary carbon atoms producing radical carbon sites in the polymer chain. Then, molecular oxygen reacts with the tertiary carbon radical to form a radical peroxy group, which then, using the neighboring hydrogen, forms a hydroperoxide. Then, the β-chain scission of PP molecules occurs by forming an olefin end group at one chain end and a peroxyl radical at the other that is eventually rearranged into a ketone group. Consequently, the degradation of PP in the presence of ozone proceeds by the formation of olefin and, with further attacks to other tertiary carbon atoms in the polymer chains, giving rise to more olefin molecule formation.

#### 2.1.4. Photocatalytic Technology for PP Degradation

The recently reviewed [34] study of the photolytic process, also called photoaging or photodegradation, is essential to understanding other strategies such as heterogeneous photocatalytic technology. Though many photochemical/UV reactions with ozone or hydrogen peroxide were carried out in some AOTs to remove organic pollutants, such as microplastics [61], the main disadvantage is the rise of harmful intermediates. This can be overcome by photocatalytic degradation. In photocatalytic degradation, organic pollutants present in wastewater are completely mineralized into carbon dioxide, water, and other non-toxic products. Nowadays, photocatalysts, which can enhance the degradation process under UV irradiation, are commonly used. The principle of photocatalysis involves the excitation of electrons from the valence band to the conduction band, thereby forming electron–hole pairs. Photogenerated electrons and holes are responsible for the reduction and oxidation reactions, respectively, occurring at the surface of the heterogeneous photocatalysts. Holes generated in the valence band react with H_2_O/OH^−^/H_2_O_2_ to form free radicals, which eventually lead to possible mineralization of organic pollutants. These species lead to an oxidizing environment through many parallel reactions, which will achieve the photodegradation of plastics. The process will result in the reduction of the plastic particle size and thus improve the suspension/solubility of plastics in the water and ultimately into the complete degradation.

Wastewater contaminated with hazardous chemicals, pesticides, phenols, chlorophenols, and other pollutants was effectively treated using photocatalysis. It also inactivates the viruses, bacteria, and protozoa residuals. Recently, photocatalysis has also been used to treat plastic waste material. To avoid the toxic by-products formed from other disposal methods, photocatalysis could be carried out to degrade the plastic waste with the help of a suitable catalyst.

Photocatalytic processes are seen as an efficient and eco-friendly method to convert plastics into value-added molecules [62]. Also, this technology has been demonstrated to be promising for PP treatment, among other plastics degradation. The sustainability, good performance, low cost, and soft conditions enforce the applicability of this strategy because it can exploit free and endless solar irradiation. Photocatalysis mineralizes contaminants to H_2_O and CO_2_ by the generation of ROS, i.e., ^•^OH, O_2_^•−^, and ^1^O_2_, along with the holes (h^+^) produced in the valence band of the semiconductor under UV-Vis irradiation.

Titanium dioxide is the most used photocatalyst because of its high oxido-reduction ability, chemical stability, high stability, cost-effectiveness, and environmental friendliness [63]. A report on the ability of TiO_2_ as a photocatalyst for the degradation of polyolefins was published as early as 1974 [64]. The use of photocatalysis for the degradation and removal of different types of pollutants, including plastic, has been studied with increasing attention, although many problems, such as an electron–hole recombination, modulation of the band gap of the semiconductors, and slow kinetics of surface reactions, still remain to be solved [58]. An important issue to be focused on in the future is the chemicals generated during the photocatalytic degradation of plastics, which have not been studied from an environmental perspective [65].

A number of semiconductor oxides, such as TiO_2_, ZrO_2_, ZnO, BiOCl, and C_3_N_4_, among others, have been successfully used as photocatalysts to degrade polymers, including PP. TiO_2_ has been recognized as the most efficient due to its excellent thermodynamical features, photogenerated carriers mobility, optical properties, non-toxicity, stability, and low cost.

A green bioinspired synthesis for C,N-TiO_2_ photocatalysts has been explored, using the mussels’ extrapallial fluid as a doping source for titania to be used as a photocatalyst for microplastic degradation. No photolytic deterioration of the MPs was observed in the reaction conditions utilized in this study, but photocatalytic degradation of primary HDPE MPs extracted from a commercial facial scrub was demonstrated. The result was evidenced by mass loss determination, degradation rate calculation, and microscope observations, among others [66]. In addition, after photocatalytic experiments, it was found that at pH = 3, hydrogen atoms were accessible as H^+^ ions. Interestingly, increasing the concentration of H^+^ enhanced the amount of hydroperoxy radical (^•^OOH), justifying the promoting degradation of the pollutant at pH 3. Photolysis at pH 3 does not result in plastic degradation due to the absence of hydroperoxides. The pH affects not just plastic breakdown but also the surface charge of TiO_2_ particles (which possess a Point of Zero Charge (PZC) of ca. 6) and hence the electrostatic attraction of microplastics to the surface of the semiconductor. At pH 3, colloidal nanoparticles of titania showed a stronger contact with MPs, leading to a faster breakdown. The degradation of HDPE MPs at low temperatures (pH 3 and 0 °C) can be explained based on the photocatalytic system’s specific properties. Microplastics, as extracted, and possessing sizes 240–725 times larger than those treated in the presence of C,N-TiO_2_, make the adsorption on the semiconductor surface impossible, unlike in other photocatalytic systems with more frequent pollutants; consequently, this makes its degradation difficult because the photocatalytic process is efficient only on the surface of the photocatalyst.

The removal of nanoplastics utilizing three distinct TiO_2_-based photocatalysts was examined to obtain fresh insights into the removal of polystyrene primary nanoplastics from aqueous solutions using UV light. The results were discussed by examining the turbidity of the suspensions. The use of the various TiO_2_ architectures resulted in substantial deterioration of the target organic polymer [67]. The most effective structure gave optimal transfer and separation of the photogenerated charge carriers, as well as the most efficient polystyrene photodegradation.

According to Asghar et al. during the photodegradation, the polyethylene coated with TiO_2_ forming the composite film (PE-TiO_2_ film) achieves a more efficient degradation rate compared to pure PE film under UV and artificial light irradiation [68]. The presence of oxidant species such as peroxydisulfate (S_2_O_8_^2−^) in the presence of TiO_2_ enhances the photocatalytic degradation of the plastic [69]. Similarly, the polystyrene (PS) TiO_2_ composite photodegradation process was faster than that of the photolysis of pure polymer samples. During this photodegradation, there is no dioxin or other component released. Bandara et al. have compared TiO_2_ and ZrO_2_ suspensions for PE and PP photocatalytic degradation under natural or simulated solar irradiation, concluding that ZrO_2_ showed higher degradation than TiO_2_ at the same experimental conditions [70].

ZnO has also been used in organic polymer photocatalytic degradation due to its suitable optical properties, excellent redox ability, great electron mobility, and non-toxicity. Tofa et al. prepared ZnO via spray pyrolysis that was used for the photocatalytic degradation of a specimen plate of low-density polyethylene (LDPE) film sized 1 cm^2^. The experiment was carried out for 175 h in a Petri dish containing the photocatalyst and deionized water. The results reveal that heterogeneous photocatalysis enhances the formation of carbonyl and vinyl groups, thus indicating the degradation of the polymeric film [71]. As a result, the photocatalytic degradation of the polymer gave rise to its oxidation, producing low-molecular-weight molecules and leading to brittleness, wrinkles, cracks, and cavities on the LDPE surface. Additionally, increasing catalyst surface area improves polymer breakdown. The use of Pt enhances more than 15% of the degradation of the plastic under visible light due to plasmon absorption and also by lowering the electron–hole recombination on ZnO [72]. Authors claim that superoxides and hydroxyl radicals formed during photocatalysis are the oxidant species responsible for plastic degradation. Uheida et al. presented a sustainable and green solution approach to eliminate microplastics using visible light by trapping low-density particles of plastics like polypropylene (PP) on glass fiber substrates, while also supporting the photocatalyst material [73]. This study shows that visible light irradiation of zinc oxide nanorods (ZnO NRs) mounted on glass fiber substrates can degrade PP microplastics floating in water in a flow-through system. Irradiating PP microplastics with visible light for two weeks resulted in a 65% reduction in average particle volume.

Other semiconductors such as Cu_x_O have been also developed via anodization for analogous scope. The anodization procedure gave rise to Cu_2_O/CuO semiconductors with varying morphologies and a bandgap of 1.6 to 2 eV [74]. Results revealed that photocatalysis using visible light irradiation was able to obtain the polymer chain scission up to 23%, six times more than the degradation achieved by photolysis. In addition, a mineralization of up to 15% was accomplished. BiOCl has been also used for the degradation of polystyrene nanoplastics by preparing polystyrene-based nano-composites with flower- and disk-shaped BiOCl nanoparticles. The photocatalytic degradation of the films under visible irradiation was tested by mechanical, morphological, and optical properties. The deterioration of the films was remarkable [75]. NiAl_2_O_4_ prepared via co-precipitation and hydrothermal methodologies was used for the degradation of commercially available polyethylene (PE) bags by photocatalysis [76]. The results obtained from FTIR analysis carried out after the degradation process in the presence of the spinel confirmed that the polyethylene sheet was degraded in 5 h showing a weight loss of ca. 12%. The degradation of a low-density polyethylene (LDPE) film was studied by using Au nanoparticles as a photocatalyst. The photoinduced degradation of the LDPE@Au nanocomposite film was higher than that of the pure LDPE film. The weight loss of LDPE@Au with 1 wt% of Au reached ca. 52% after 240 h of solar light irradiation, compared with the 9% photodegradation of the polymer in the absence of Au. The solid maintains its activity even after five consecutive cycles of photocatalytic runs [77].

Photocatalytic degradation of plastic materials can be performed in an integrated process in which contemporaneously to the oxidation of polymers dispersed in water also hydrogen is formed. The next paragraph will discuss this interesting novel approach by considering polypropylene as the polymer to be degraded. It is worth noting that, however, the number of scientific reports in this field is increasing, there are currently still few publications dedicated to this topic, and, in particular, those reporting the use of PP as the substrate to be oxidized simultaneously with the generation of hydrogen.

## 3. Polypropylene (PP) Waste as Source of Green Hydrogen in Photoreforming

Plastic recycling has been handled in three ways: depolymerization, partial oxidation, or cracking. Depolymerization via reversible synthesis reactions (i.e., alcoholysis, glycolysis, and hydrolysis) works well for polyamides and polyesters (e.g., nylons and polyethylene terephthalate (PET)), but requires harsh conditions as high temperature and pressure [78]; nonetheless, it is ineffective for polypropylene, and, consequently, the oxidative vias in coordination with the obtaining of H_2_ in the same process seems particularly appealing.

**Figure 9:**
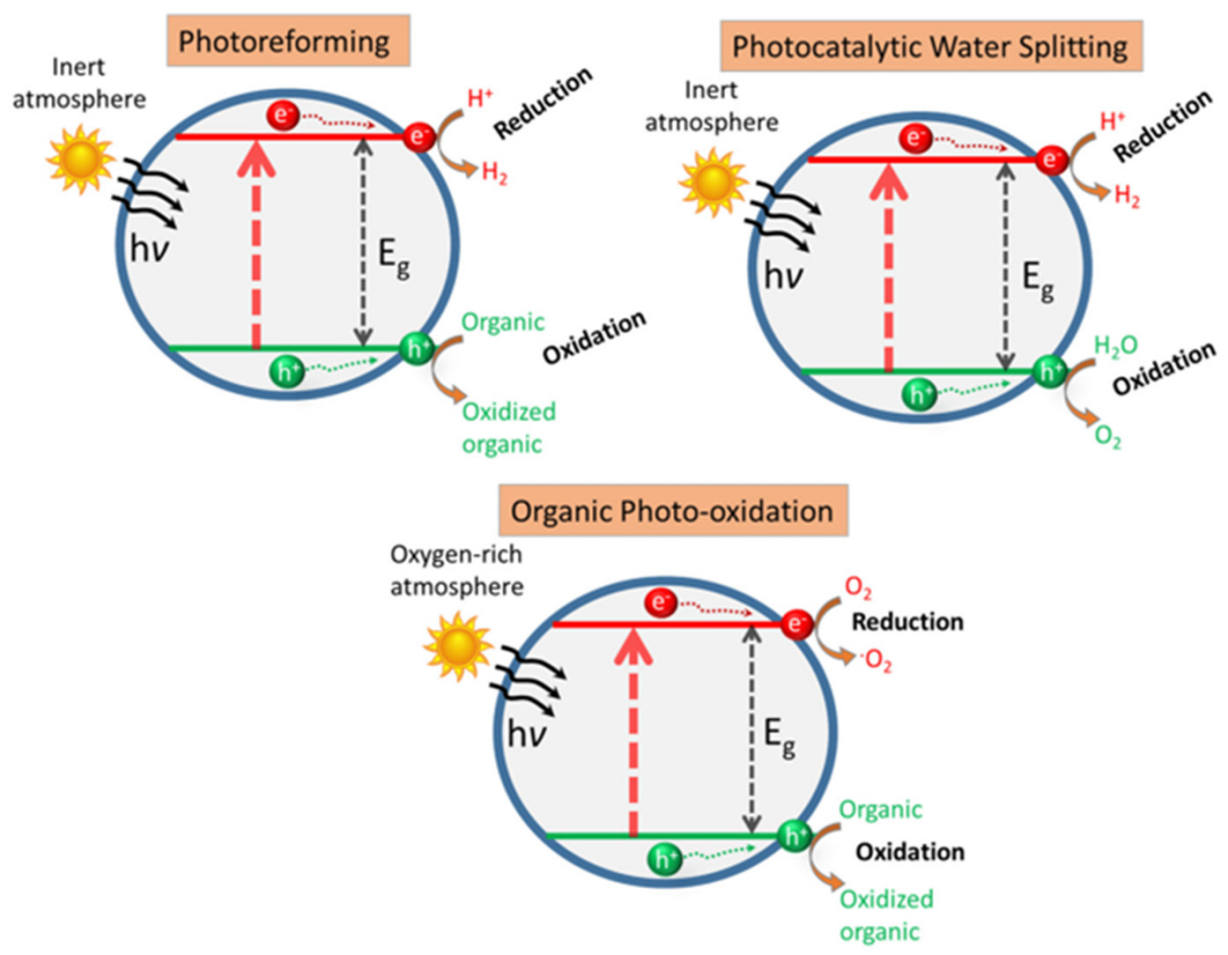
Mechanism of the photoreforming reaction along with the photocatalytic water splitting and the oxidation of an organic molecule in the presence of oxygen; from [81].

Hydrogen production by photocatalysis in the presence of light is a versatile and environmentally benign manner to obtain energy. Photoreforming (see Figure 9) is the reaction able to transform an organic, which can be a waste, into a valuable chemical and at the same time to obtain clean H_2_ fuel [79]. Hydrogen can be produced at room temperature and atmospheric pressure by a simple, efficient, low-cost, and sustainable process, with the use of a heterogeneous photocatalyst using a waste material that can be biomass or plastic, light, and water [80]. Photoreforming involves the splitting of water to generate H_2_ by a reduction reaction and the simultaneous oxidation of an organic species to obtain other molecules with higher added value or, simply, to completely oxidize (mineralize) organics to CO_2_ and H_2_O.

As we have mentioned before, PP possesses C-C bonds in its backbone and it is susceptible to light-induced oxidative degradation, which is the most effective abiotic degradation route of plastic in outdoor environments [82] involving a radical mechanism. The initiatory degradation step, in which light leads to cleavage of the chemical bonds in the backbone chain of the polymer, is favored by the presence in the chain of unsaturated chromophore groups that can absorb light giving rise to the formation of free radicals [83]. Unfortunately, this mechanism cannot be applied to all synthetic polymers as most of them, like PP, do not possess chromophores capable of absorbing UV light. Consequently, in the case of PP, which does not present unsaturated chromophores or double bonds it is more difficult to generate free radicals [84]. However, in the case of photoreforming, which must be considered as a photocatalytic process there is always the formation of radical species like ^•^OH, O_2_^•−^ (see Section 2.1.4) that are capable of initiating the degradation of the polymer chain as already discussed in Section 2.1.1 and Section 2.1.3 (see also Figure 6, Figure 7 and Figure 8). PP has tertiary carbon atoms while PE has secondary carbon atoms; for this reason, PP has lower stability, which makes it more susceptible to abiotic attacks. However, regarding the mechanism of degradation, it is quite similar for PP and PE, leading to low-molecular-weight fragments. In addition to the decrease in molecular weight, new functional groups such as carbonyl groups are formed, which leads to products, including pentene, 2-methyl-1-pentene, and 2,4-dimethyl-1-heptene. Plastics containing a C-C bond in the main structure can be used as hole scavengers in the process of photoreforming, giving rise to oxidized products, possibly into fine chemicals with the concomitant formation of H_2_ gas by the reduction of H_2_O at room temperature. This reaction has been reported to occur with some semiconductor oxides [85].

Recently, Liang et al. have reviewed the new results on the conversion of plastic into fuels, fine chemicals, and materials [86], concluding that the photocatalytic processes are more environmentally friendly and sustainable than thermo-catalysis under harsh conditions. These technologies for plastic upcycling are still in their early stages and there is still a huge space to improve the efficiency for practical production and real applications. Particularly, very limited work has been devoted to the upcycling of plastics in photoreforming reactions. The literature is very limited in this area and it is in great augment.

Polyethylene terephthalate was used as a hole scavenger in photoreforming using SiC-g-C_3_N_4_ composites as photocatalysts. The presence of the heterojunction produced by small amounts of g-C_3_N_4_ on the surface of SiC enhances the separation of the photoproduced electron and holes and yields H_2_ with a rate of 18 μmol per gram of catalysts and hour of reaction. The polymer oxidation gave intermediates such as ethylene glycol, which further enhanced the photoreforming effectiveness. It is worth noting the strong basicity of the suspension (pH = 13) that was essential to pre-treat the polymer and that favors the interaction between the reactants and the surface of the heterogeneous photocatalyst [87].

Xu et al. have treated plastic PE bags, PP boxes, and PET bottles, which were mineralized to CO and H_2_, i.e., syngas, in the presence of H_2_O by the Co-Ga_2_O_3_ nanosheets. The H_2_ and CO formation rates were approx. 648 and 158 μmol g^−1^ h^−1^ ca. double the amount of that obtained with bare Ga_2_O_3_. The weight losses of PE bags, PP boxes, and PET bottles were ca. 81, 78, and 72% after 48 h irradiation in the presence of Co-Ga_2_O_3_ [88]. Cao et al. have developed an effective photoreforming process in which the H_2_ production was combined with PET microplastic degradation in the presence of MXene/Zn_x_Cd_1−x_S [89]. The highest photocatalytic H_2_ evolution rate reached 14 mmol g^−1^·h^−1^ in alkaline polymer solution. PET was transformed into glycolate, acetate, and ethanol. C_3_N_4_ was also tested for alkaline PET solution degradation giving rise to a H_2_ evolution rate of 600 mmol g^−1^ h^−1^ [90]. A Z-scheme heterostructure of V-substituted phosphomolybdic acid and C_3_N_4_ has also been tested in the upcycling of various plastics. The optimal composite exhibits a remarkable formic acid production rate of 55 μmol g^−1^·h^−1^ for the upcycling of polyethylene, which is 262-fold higher than that of pristine C_3_N_4_ [91]. In a slightly different approach, Jiao et al. propose the selective conversion of waste plastics, such as polyethylene, polypropylene, and PVC, in the presence of Nb_2_O_5_. Polyethylene was photodegraded completely into CO_2_, which was then photoreduced into acetic acid [92].

## 4. Conclusions

Inefficient disposal of plastic materials represents a serious environmental problem; therefore, scientific studies regarding possible strategies dedicated to their treatments can contribute to mitigating the pollution linked to the uncontrolled release of these materials into the environment. The slow kinetics of the natural degradation of polymers and in particular of the most used ones, such as polypropylene (PP), makes it necessary to use advanced oxidation processes, which are technologies capable of offering a strategy for the treatment of this type of waste. Through these processes, various organic pollutants and recalcitrant chemicals are effectively removed from both water and wastewater. Processes such as ozonation, catalysis in the presence of UV radiation, hydrogen peroxide, and/or heterogeneous photocatalytic processes generate intermediates that can act on the degradation of polymers and implement their introduction in a circular economy framework. This mini-review provides an overview of the strategies that can be implemented in this framework. Emerging aspects for the treatment of these recalcitrant pollutants are examined with particular attention to the degradation of polypropylene, chosen as an example of a material widely present in the environment as a recalcitrant pollutant when dispersed in soil or water. In fact, the presence of micro and nanoplastics is well known as a serious cause of threat to human health and the environment. The mini-review describes economical and efficient methods for polymer degradation. The chemical recycling of polypropylene (PP) can be addressed through its depolymerization to obtain polymers with a lower molecular weight that may be suitable for returning to the original material preparation cycle or which could be used as fuels to support the process itself. This overview gives an account of the results obtained by applying these technologies and studying the different operating parameters.

## Data Availability

No new data were created.
